# Multiple Aggregates and Aggresomes of C-Terminal Truncated Human αA-Crystallins in Mammalian Cells and Protection by αB-Crystallin

**DOI:** 10.1371/journal.pone.0019876

**Published:** 2011-05-12

**Authors:** Ilangovan Raju, Anbarasu Kumarasamy, Edathara C. Abraham

**Affiliations:** 1 Department of Biochemistry and Molecular Biology, University of Arkansas for Medical Sciences, Little Rock, Arkansas, United States of America; 2 Department of Biotechnology, Bharathidasan University, Tiruchirapalli, India; University of Kent, United Kingdom

## Abstract

**Background:**

Cleavage of 11 (αA162), 5 (αA168) and 1 (αA172) residues from the C-terminus of αA-crystallin creates structurally and functionally different proteins. The formation of these post-translationally modified αA-crystallins is enhanced in diabetes. In the present study, the fate of the truncated αA-crystallins expressed in living mammalian cells in the presence and absence of native αA- or αB-crystallin has been studied by laser scanning confocal microscopy (LSM).

**Methodology/Principal Findings:**

YFP tagged αAwt, αA162, αA168 and αA172, were individually transfected or co-transfected with CFP tagged αAwt or αBwt, expressed in HeLa cells and studied by LSM. Difference in protein aggregation was not caused by different level of α-crystallin expression because Western blotting results showed nearly same level of expression of the various α-crystallins. The FRET-acceptor photo-bleaching protocol was followed to study *in situ* protein-protein interaction. αA172 interacted with αAwt and αBwt better than αA168 and αA162, interaction of αBwt being two-fold stronger than that of αAwt. Furthermore, aggresomes were detected in cells individually expressing αA162 and αA168 constructs and co-expression with αBwt significantly sequestered the aggresomes. There was no sequestration of aggresomes with αAwt co-expression with the truncated constructs, αA162 and αA168. Double immunocytochemistry technique was used for co-localization of γ-tubulin with αA-crystallin to demonstrate the perinuclear aggregates were aggresomes.

**Conclusions/Significance:**

αA172 showed the strongest interaction with both αAwt and αBwt. Native αB-crystallin provided protection to partially unfolded truncated αA-crystallins whereas native αA-crystallin did not. Aggresomes were detected in cells expressing αA162 and αA168 and αBwt co-expression with these constructs diminished the aggresome formation. Co-localization of γ-tubulin in perinuclear aggregates validates for aggresomes.

## Introduction

A major protein of the vertebrate eye lens, namely α-crystallin, consists of two homologous 20 kDa subunits, namely αA- and αB-crystallins [1]–[3]. These two proteins are members of the small heat-shock protein (sHsp) family and have the ability to operate as molecular chaperones by binding to partially unfolded target proteins and preventing them from aggregation [4]–[7]. The C-terminal extension and the predominantly hydrophilic flexible C-terminal tail of αA-crystallin play a vital role in the oligomerization [8], [9] as well as for ensuring solubility of the protein assemblies formed with target proteins.

Post-translational modifications of lens crystallins are believed to play a major role in the development of human senile cataract. Cleavage of amino acid residues at specific sites in the C-terminal end of αA-crystallin constitutes the major form of modification that leads to structural and functional changes in this sHsp/molecular chaperone [10]–[16]. In human αA-crystallin, 13 cleavage sites have been identified and the residues 162, 168 and 172 being the major ones [16]. Cleavage of serine from the C-terminus, which forms truncated αA172, is the most prevalent form of modification that occurs in human eye lens crystallins [10], [15], [16]. Our earlier studies have shown increased formation of αA172 in diabetic human lenses; the total level of αA172 increased from about 30% in non-diabetic lenses to about 50% in diabetic lenses [16]. Cleavage of 1, 5, and 11 residues showed diverse effects on oligomerization and chaperone function [17]. Chaperone activity of αA172 was 28–46% higher than that of αAwt and the oligomeric size was increased by 12% [17]. On the other hand, αA168 and αAwt had similar chaperone activity and molecular mass whereas αA162 behaved quite differently by showing 80–100% decrease in chaperone activity and 42% decrease in molecular mass. However, it should be emphasized that these results were obtained by studying homoaggregates, but, in human lenses they may exist as homoaggregates as well as heteroaggregates in association with native αA-crystallin and/or αB-crystallin. As heteroaggregates, the truncated αA-crystallins are expected to behave differently. The ability to associate with native αA- or αB-crystallin is dictated by the strength of the interactions between them. In a previous *in vitro* study with recombinant αBwt, αAwt and the C-terminal truncated αA-crystallins and by utilizing fluorescent chemical probes in fluorescence resonance energy transfer (FRET) analysis, we have observed C-terminal truncation affecting interaction with αAwt and αBwt [18]. However, mapping the interactions in living mammalian cells has not been done before. In addition, the present study was aimed to show, whether truncated αA-crystallins tend to aggregate in living cells and, if so, will co-expression with either αAwt or αBwt suppress aggregation? The present study also showed whether cleavage of the C-terminal residues of αA-crystallin affects its interaction with native αA- and αB-crystallins in mammalian cells.

Aggresomes are spherical or ribbon like structures localized in the perinuclear region. Protein quality control systems, such as molecular chaperones and ubiquitin-proteasome system (UPS) degrade or refold the abnormal proteins and prevents the toxic accumulation of small protein aggregates. However, when the protein quality control system is overwhelmed or evaded, the resulting small aggregates are dispersed throughout the cell and they are actively cleared via transport to intracellular inclusion bodies (IBs). These IBs are termed aggresomes or aggresome-like inclusions. These structures are conserved from yeast to mammalian cells and act as storage bins for protein aggregates [19]–[21]. The formation of aggresomes is believed to serve as cytoprotective function by refolding or degradation of unfolded or misfolded proteins [22] and they are produced around the microtubule organizing center (MTOC) for degradation [19]. In this paper, we have demonstrated aggresomes are evident in αA162 and αA168 individually expressing constructs and in co-expression of αAwt with these truncated constructs. Co-expression of αBwt with these truncated constructs significantly diminished the aggresome formation.

## Materials and Methods

### Materials

The Cyan (pAmCyan1-Cl or CFP) and Yellow (pZsYellow1-Cl or YFP) expression vectors were obtained from Clonetech ( Palo Alto, CA ), HeLa cells were obtained from the American Type Culture Collection (ATCC) (Manassas, VA), Plasmid DNA extraction kits (Qiagen, Valencia, CA) cell culture medium, fetal bovine serum (FBS), Lipofectamine 2000, Penicillin/Streptomycin (Invitrogen, Rockville, MD), restriction enzymes were from New England BioLabs Inc. (Ipswich, MA) and T4 DNA Ligase was from Promega, (Madison, WI).

### Construction of CFP and YFP-tagged α-crystallins vectors

The full-length human αB- crystallin wild-type, αA- crystallin wild-type and the C-terminal truncated αA-crystallins (αA162, αA168 and αA172) genes were PCR amplified using the appropriate primers containing restriction sites, *Xho* I and *Hind* III and cloned into the C-terminal end of the mammalian expression vectors, pAmCyan1-C1 (CFP) or pZsYellow1-C1 (YFP) driven by CMV promoter. In the present study, both human αBwt and αAwt were sub-cloned into the CFP vector for the expression of crystallin genes in cyan color and αAwt, αA172, αA168 and αA162 were sub-cloned into the YFP vector for their expression in yellow color. All the constructs were confirmed by restriction digestion analysis and sequenced at the UAMS DNA sequencing core facility.

### Cell culture and transfection

HeLa cells were cultured in MEM medium (Invitrogen, Carlsbad, CA) supplemented with 10% FBS and penicillin/streptomycin (100 µg/ml), at 37°C in 5% CO_2_ humidified chamber. About 1.0×10^5^ cells / ml were seeded into each 35 mm, sterile glass bottomed single well poly-d-lysine treated plates (MetTek Corporation, Ashland, MA, USA) and cultured in 2 ml of growth medium for transient transfection. The overnight adherent cells were transfected with Lipofectamine 2000 (Invitrogen, Rockville, MD) according to the manufacture's protocol. Briefly, each well was transfected alone or co-transfected with total 2 µg/well of pAmCyan1-C1 (CFP), and/or pZsYellow1-C1 (YFP) plasmids encoding the respective crystallin gene along with 5 µl of Lipofectamine 2000. After 6 h, transfected medium was removed and replaced with fresh medium was containing 10% FBS. After 48 h transfection, cells were examined for laser scanning confocal microscopic study. Transfected cells showing aggregates were typically counted at ×40 magnification. Fields were randomly chosen and about 300 cells were counted per experiment and repeated at least three times and counts were blindly performed.

### Western blotting for αA- and αB-crystallins expressed in HeLa cells

After 48 hours transfection, cells were lysed with lysis buffer containing 50 mM Tris-HCl (pH 7.4), 150 mM NaCl, 0.02% sodium azide, 0.1% SDS, 1% NP-40, 0.5% sodium deoxycholate and 0.1 mM EDTA supplemented with cock-tail protease inhibitors and 3 M urea. Further, cells were sonicated and the protein concentration was measured by BCA assay method. For each sample, 5 µg of protein was loaded into 12% SDS-PAGE and electroblotted to nitrocellulose membrane. The blots were blocked with 5% non-fat dry milk prepared in TBST (Tris-buffered saline supplemented with 0.1% Tween 20) and subsequently incubated with primary antibody for αA-crystallin (monoclonal, Abcam, 1∶ 2000), αB-crystallin (rabbit polyclonal, Abcam, 1∶2000) for one hour at room temperature. Blots were washed with TBST for three times and incubated with appropriate HRP-conjugated secondary antibodies (1 in 5000, Santa Cruz Biotechnology Inc, CA) for one hour at room temperature. Enhanced chemiluminescence substrate was used and the signal was detected by exposing the blots on films. For loading control, blots were stripped with Restore Western Blot stripping buffer (Thermo Scientific Inc, IL) and re-probed with a rabbit polyclonal antibody against β-actin (Abcam, 1∶ 10000) for 1 hour at room temperature.

### Immunofluorescence microscopy

Cells were grown on 35-mm cover glass bottom dishes. After 48 hours transfection, cells were washed with PBS, fixed with 4% paraformaldehyde for 20 minutes at room temperature (RT) and permeabilized with 0.5% Triton X-100 for 10 minutes at RT. Cells were blocked with 3% Normal Goat Serum (NGS) for one hour at RT and labeled with primary antibody for αA-crystallin in 3% NGS (1 in 500) for overnight at 4°C and subsequently incubated with Alexa Fluor 594 goat-anti-mouse IgG secondary antibody (Molecular Probes) diluted in 3% NGS (1 in 500) for one hour at RT and washed with PBS. For double immunofluorescence, cells were fixed with 4% paraformaldehyde and permeabilized in 0.5% Triton X-100 and blocked with 10% normal goat serum (NGS) and simultaneously incubated with the two primary antibodies, αA-crystallin (Mouse monoclonal, 1 ∶ 500) and γ-tubulin (Rabbit polyclonal, Abcam 1∶ 500) diluted in 5% NGS for overnight at 4°C and washed with PBS for five times. The cells were then stained with Alexa Fluor 594 Goat anti-mouse (1∶500) and Alexa Fluor 488 Goat anti-rabbit (1∶500) diluted in 5% NGS for one hour at RT and washed with PBS for three times. Hoechst 33342 (Molecular Probes) was used to stain the nuclei. The images were acquired with an LSM 510 Meta Carl Zeiss Confocal microscope at ×63 objective and analyzed using AIM Imaging Software.

### Laser scanning confocal microscope studies

A Zeiss Meta LSM 510 Laser Scanning Microscope (Carl Zeiss Inc., Thornwood, NY) with ×63 oil-immersion objective (plan Apochromat, NA 1.4) (University of Arkansas for Medical Sciences core facility) was utilized. To visualize CFP and YFP fluorescence, cells expressing fluorescent proteins were excited with appropriate laser beam and filtered with both dichromatic band pass filters, captured at 12 bit 512×512 multi-track channel images with CCD cameras with the following configurations: for CFP channel, the cells were excited with 458 nm filter by argon-ion laser and the emission intensity was collected using band pass (BP) 475–525 nm filters and for YFP channel, the cells were excited with 514 nm filter by argon-ion laser and the emission intensity was collected using BP 530–600 nm filters. Both the CFP and YFP was excited using argon-ion laser at 25 mW, 2.0 and 0.5% exposure respectively. All images were taken at room temperature.

### FRET analysis by live acceptor photobleaching method

The acceptor photobleaching method is one of the accurate methods available to determine the interaction between two proteins based on the increased intensity of donor fluorescence at the time of acceptor bleaching. In this method, the acceptor fluorescence was bleached with the help of high intensity argon laser light (100% exposure at 514 beam). A series of pre-bleaching and post-bleaching donor and acceptor signal collecting protocols were automated for the acquisition of pre-bleach and post-bleach images and noted the increased level of donor intensities due to de-quenching and decreased level of acceptor signal due to photo-bleaching. The increased donor (CFP) fluorescence intensity and decreased acceptor (YFP) fluorescence intensity is the sign for the occurrence of protein-protein interaction. The FRET efficiency was calculated based on ten images taken from each construct examined and each experimental condition was performed 3 times and values were averaged. The FRET efficiency (E) was calculated by: E = 1−(Ipre/Ipost)×100%, where Ipre is pre-bleach fluorescence intensity and Ipost is post-bleach fluorescence intensity.

### Aggresome staining

HeLa cells were grown on glass bottom 35 mm dishes and transfected with YFP-tagged αAwt, αA162, αA168 and αA172 constructs individually and or co-transfection with CFP-tagged αA-wt or αB-wt. After 48 h transfection, cells were fixed in 4% paraformaldehyde for 30 minutes at room temperature (RT) and permeabilized with 0.5% Triton X-100 in 1× assay buffer for 30 minutes on ice. Cells were washed with 1× assay buffer for two times and stained with ProteoStat Aggresome dye (Enzo Life Sciences, PA) for 30 minutes at RT and washed with 1× assay buffer. The stained cells were examined with an LSM 510 Meta Confocal microscope and images were captured at ×63 objective with red filter.

### Statistical analysis

A two-tailed Student's t-test was used to calculate the significance between the wild-type and the truncated αA-crystallin groups. The p value<0.05 was considered as significant.

## Results

### Individually expressed αAwt and its C-terminal residues cleaved proteins in HeLa cells

**Figure 1:**
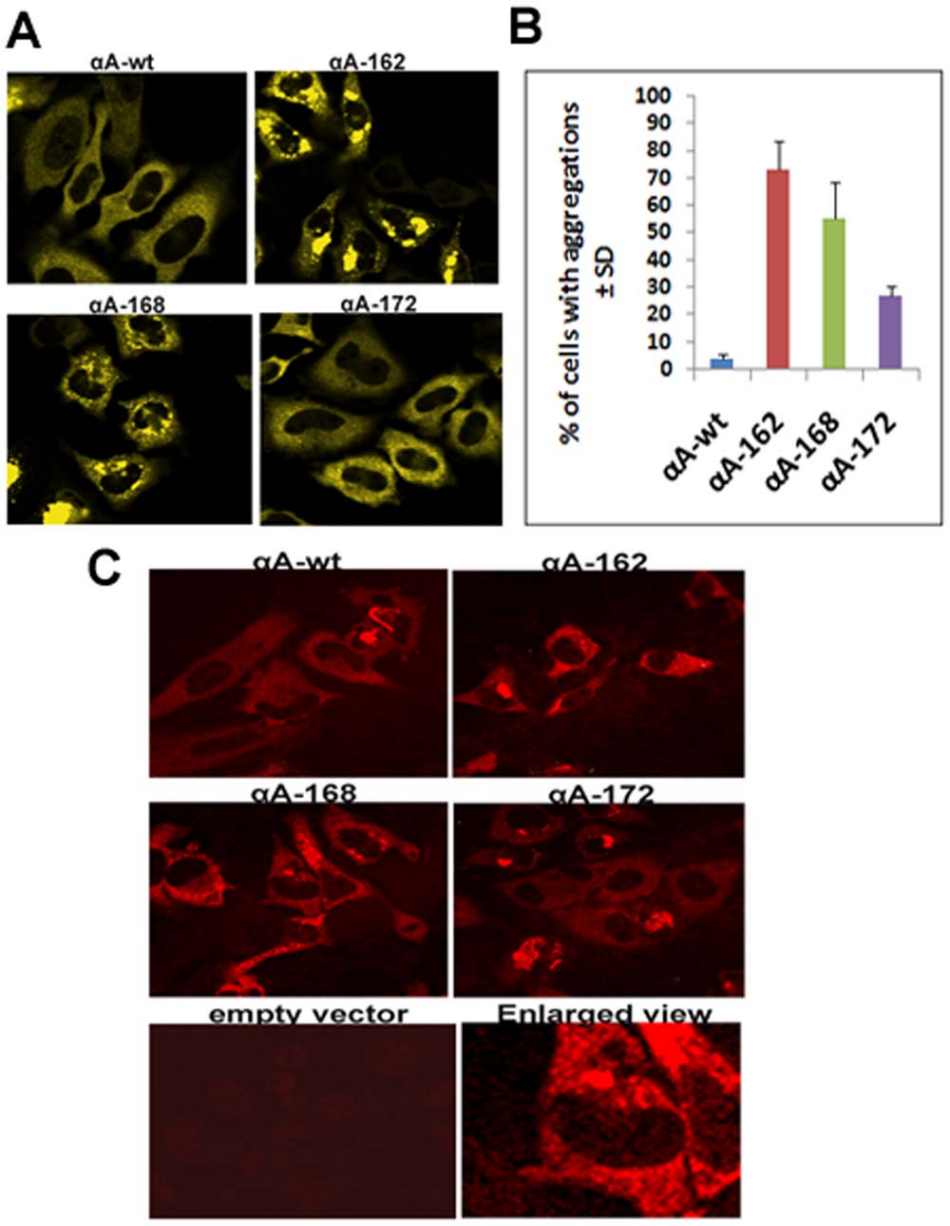
Indidivudal expression of YFP-tagged αA-Crystallin wild-type and truncated constructs in HeLa cells. ***A:*** *LSM confocal images of HeLa cells expressing αA-wt and truncated constructs showing intracellular aggregates*. Cells expressing withYFPαA162 and YFPαA-168 showing aggregates but not in αA-wt and αA172 transfected cells. YFP was excited at 514 nm and the images were collected by BP 530–600 nm filter. ***B:*** *Bar diagram illustrating % of cells containing aggregation (as illustrated in* *Fig. 1A* *)*. Randomly selected fields of 50 cells were counted and % of cells containing aggregation was calculated for each group. The values of all the truncated constructs are statistically significant at p<0.05 (t-test) compared to the αAwt group. ***C:*** *Immunofluorescence analysis of αA-crystallin over expression in HeLa cells*. αA-crystallin is abundantly localized in the cytoplasm, however, nuclear localization as in the form of foci were seen in αAwt, αA162 and αA168 individually expressing cells. In cells transfected with empty vector, there was no staining indicates that there was no endogenous expression of αA-crystallin. Cells containing aggregates were stained intensely in αA162 and αA168 expressing cells. An enlarged view from αA168 expressing cells shows nuclear foci (bottom right panel) and stained cytoplasmic aggregates. The images are representative of four similar images obtained in three independent experiments.

**Figure 2:**
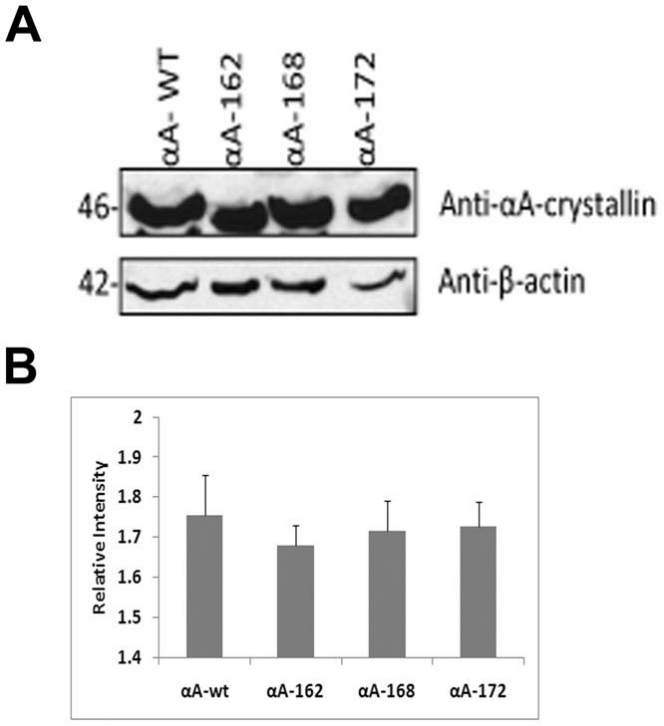
Level of expression of the wild-type and truncated αA-Crystallin constructs in HeLa cells. ***A:*** *Western blot analysis of individual expression of αA- constructs in HeLa cells*. Cells were transfected with a total of 2 µg of YFPαAwt, YFPαA162, YFPαA168 and YFPαA172 constructs. After 48 hours transfection, cells were lysed and subjected to immunoblot and probed with anti-αA-Crystallin antibody. This antibody is specific for αAwt and the truncated αA-crystallins, which recognizes a 46 kDa (the mass of αA-crystallin+YFP) protein. The same blot was stripped and re-probed with anti-β-actin for loading controls. ***B:*** *Quantitative data for western blot analysis as in* *Fig. 2A*. Quantitation of αA-Crystallin wild-type and truncated constructs normalized against β-Actin was determined with NIH Image J Software. The levels were averaged over three independent experiments and plotted. The values are means ± Standard Deviation.

HeLa cells were transfected with YFP-DNA constructs for αAwt and the three truncated αA-crystallins individually and laser scanning confocal microscopic (LSM) images were taken after 48 h (Fig. 1A). LSM images of the CFP vector alone and YFP vector alone showed full expression of each vector in both the nucleus and the cytoplasm with no evidence of aggregation (data not shown). Expression of YFP-αAwt and YFP-truncated αA-crystallins was mostly confined to the cytoplasm. However, the immunostained cells show that nuclear localization in the form of foci is evident in αAwt and the truncated constructs over-expressed cells (Fig. 1C). In addition, there was an evidence for the presence of protein aggregates predominantly in the cells expressing αA168 and αA162 and to a lesser extent in cells expressing αA172. In the immunofluorescence microscopic images, these aggregates were stained intensely than the diffuse staining pattern seen in αAwt and αA172 expressing cells (Fig. 1C). Moreover, the shape of the cells expressing these truncated αA-crystallins appeared abnormal and distorted. When αAwt, αA172, αA168, and αA162 were expressed individually, nearly 3, 27, 55 and 74% cells, respectively, had significant level of aggregates (Fig. 1B). Western blotting with anti-αA antibody showed nearly equal level of expression of αA-wt and each of the truncated αA-crystallin (Fig. 2). Thus, the difference in the levels of protein aggregation was apparently not due to different level of expression of the various forms of αA-crystallin. Furthermore, the immunofluorescence results suggest that there was no endogenous expression of αA-crystallin in empty vector transfected HeLa cells (Fig. 1C).

### Co-expression of CFP-αAwt with YFP-αAwt and YFP-truncated αA-crystallins

**Figure 3:**
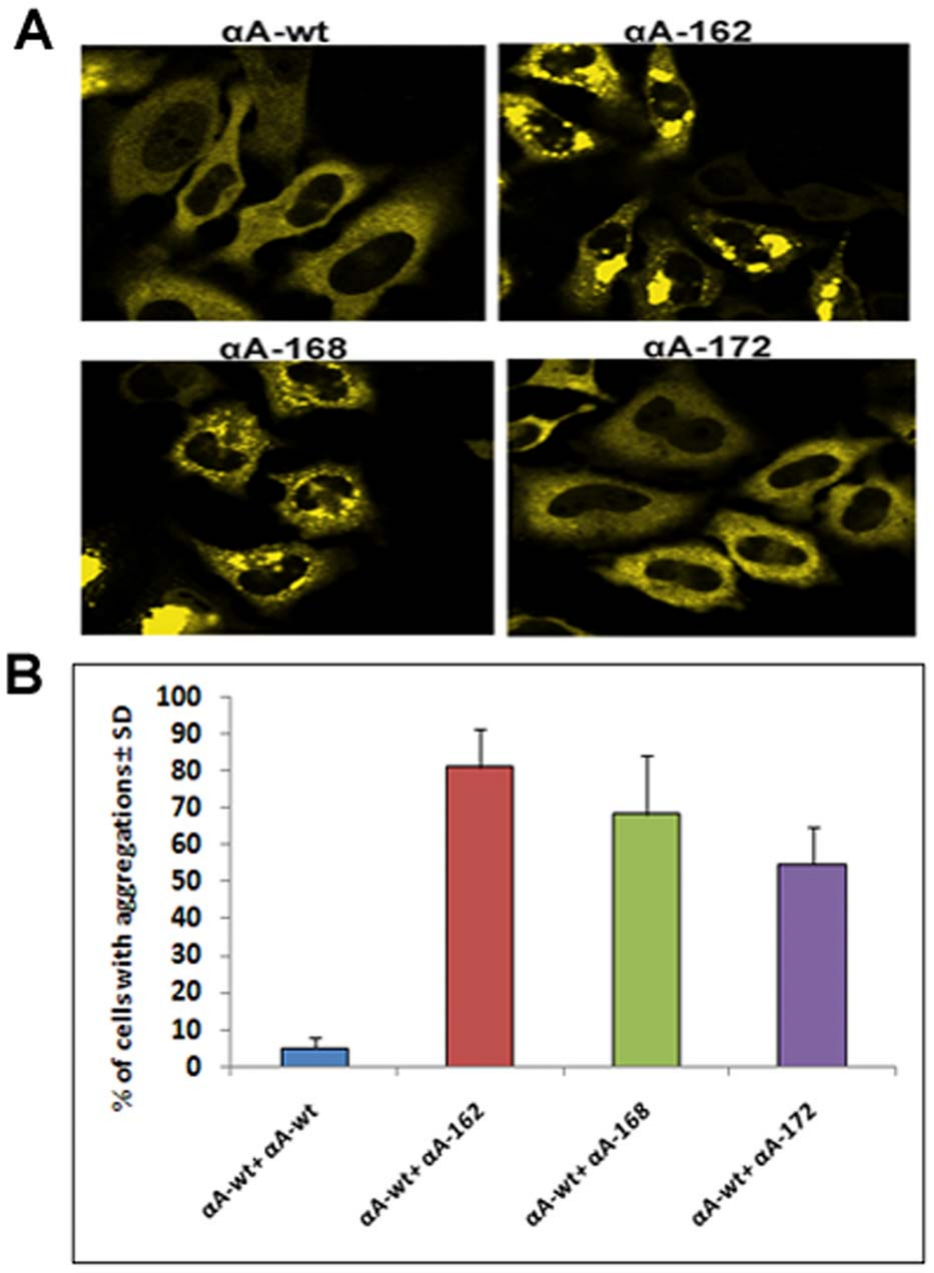
Co-expression of CFP-tagged αA-Crystallin wild-type with YFP-tagged αA-Crystallin wild-type and truncated constructs. ***A:*** *LSM confocal images of HeLa cells*. Cells co-transfected with CFPαAwt with YFPαAwt and with each of the YFP-C-terminal truncated αA-crystallins; CFPαAwt/YFPαAwt, CFPαAwt/YFPαA172, CFPαAwt/YFPαA168, and CFPαAwt/YFPαA162. CFP was excited at 458 nm and images were collected by BP 475–525 nm filter and YFP was excited at 514 nm and the images were collected by BP 530–600 nm filter. ***B:*** *Bar diagram showing % of cells with aggregations*. The values were determined as in fig. 1B. The values for all the truncated constructs are significant at p<0.05 Vs αAwt+αAwt groups.

**Figure 4:**
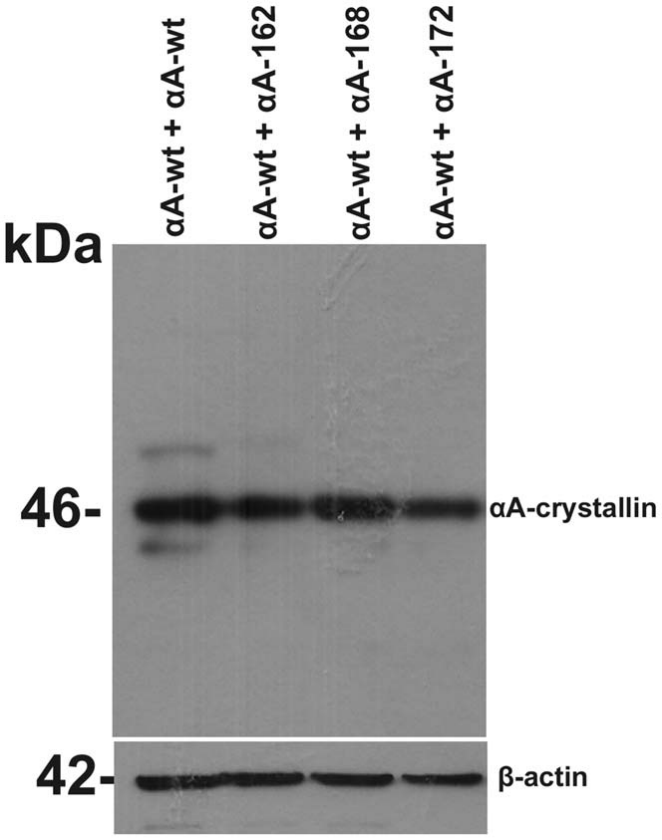
Level of expression of the various αA-crystallins in HeLa cells when they were co-expressed with αAwt. Western blotting was probed with anti-αA antibody. The details are as in Fig. 2.

HeLa cells were co-transfected with CFP-αAwt and each of the YFP- αA-truncated and the images were collected after 48 h (Fig. 3A). Co-expression of CFP and YFP vectors alone in HeLa cells resulted in the presence of both the vectors in the cytoplasm as well as the nucleus with no apparent aggregation (data not shown). As expected, protein expression was confined to the cytoplasm only when CFP- and YFP-tagged proteins were co-expressed (Fig. 3A). Co-expression with αAwt has not improved the appearance of the cells and has not decreased protein aggregation within the cells. Significant protein aggregation was seen in the cells expressing αA162, αA168, and αA172, as shown by 81, 68 and 54% of the cells, respectively, having significant protein aggregates (Fig. 3B). This shows actual increase in protein aggregation, probably due to co-aggregation of truncated αA-crystallins with αAwt. Western blotting with anti-αA antibody showed nearly equal level of total αA expression (anti-αA antibody does not distinguish between αAwt and the truncated forms) (Fig. 4).

### Co-expression of CFPαBwt with YFPαAwt and YFP-truncated αA-crystallins

**Figure 5:**
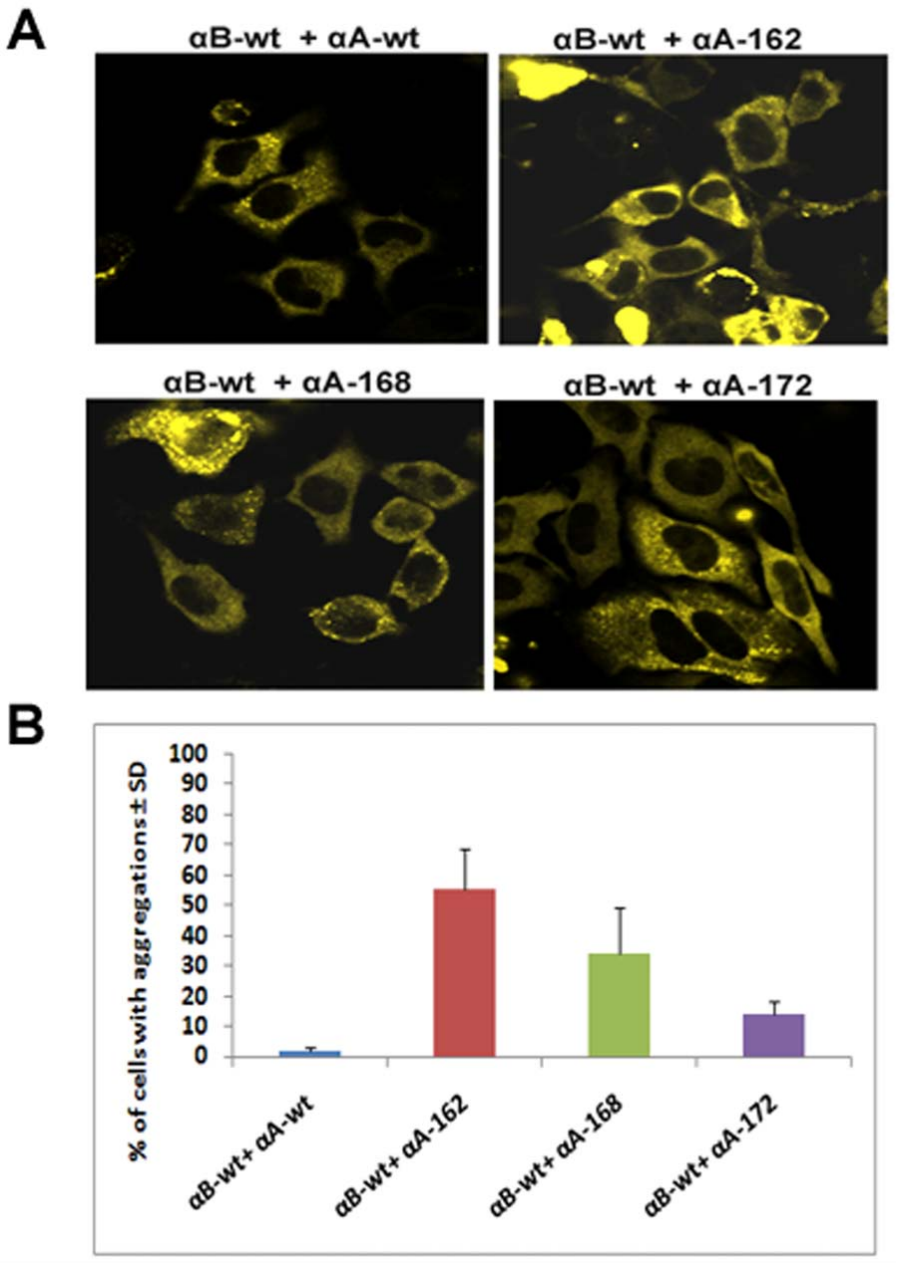
Co-expression of CFP-tagged αB-Crystallin wild-type with YFP-tagged αA-Crystallin wild-type and truncated constructs. ***A:*** *LSM confocal images of human CFPαBwt co-expressed with YFPαAwt and YFP-C-terminal truncated αA-crystallins in HeLa cells*. CFP was excited at 458 nm and the images were collected by BP 475–525 nm filter, YFP was excited at 514 nm and the images were collected by BP 530–600 nm filter. ***B:*** *Bar diagram showing the percent of cells containing aggregates (from* *fig. 5A* *)*. The values were determined as in fig. 1B. The mean values are statistically significant at p<0.05 for αBwt+αA162 and αBwt+αA172 Vs αBwt+αAwt groups.

**Figure 6:**
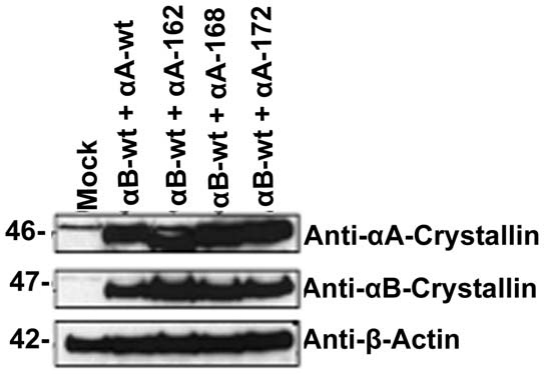
Level of expression of the various αA-crystallins when they were co-expressed with αBwt. Western blotting was probed with anti-αB antibody and anti-αA antibody. The details are as in Fig. 2. The results validate the transfection efficiency of each of the pairing of αA and αB constructs is nearly equal. There is a non-specific immunoreactive band was observed in mock control cells, lane 1.

HeLa cells were transfected with pairs of CFPαBwt and YFPαAwt and all the truncated αA-crystallins and the images were collected after 48 h (Fig. 5A). By co-expression with αB, there was clear indication of improvement in cell morphology even in the presence of truncated αA- crystallins like αA168 and αA162. Cells carrying protein aggregates also decreased substantially as indicated by a decrease in the cells containing aggregates to 1, 4, 34, and 55%, respectively for CFPαB/YFPαAwt, CFPαBwt/YFPαA172, CFPαBwt/YFPαA168 and CFPαBwt/YFPαA162 (Fig. 5B). Thus, the presence of αB-crystallin has shown significant inhibition of protein aggregation, although cells expressing αA162 still remained vulnerable to aggregation. Western blotting with both anti-αA and anti-αB antibodies showed almost the same level of expression of the various αA-crystallins and αBwt (Fig. 6).

### Results of *in situ* FRET studies by LSM image analysis for homologous and heterologous interactions

**Figure 7:**
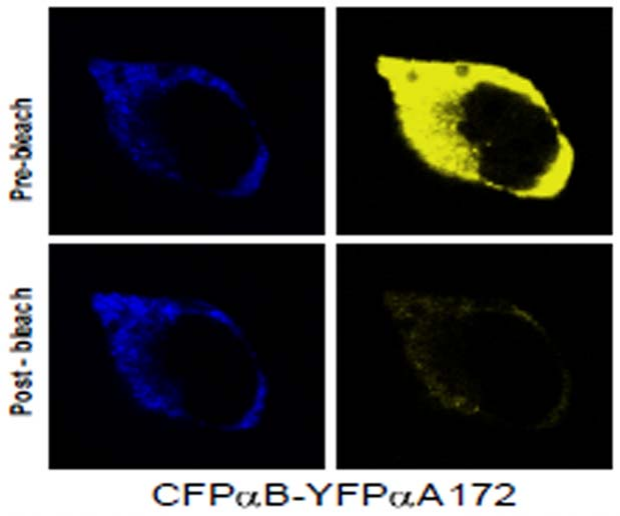
Illustration of the acceptor photobleaching method for determining FRET efficieny. In this example, CFPαBwt (donor) was co-expressed with YFPαA172 (acceptor). The acceptor fluorescence was bleached by high intensity argon laser light. This resulted in an increase in donor fluorescence intensity and a decrease in acceptor fluorescence.

**Figure 8:**
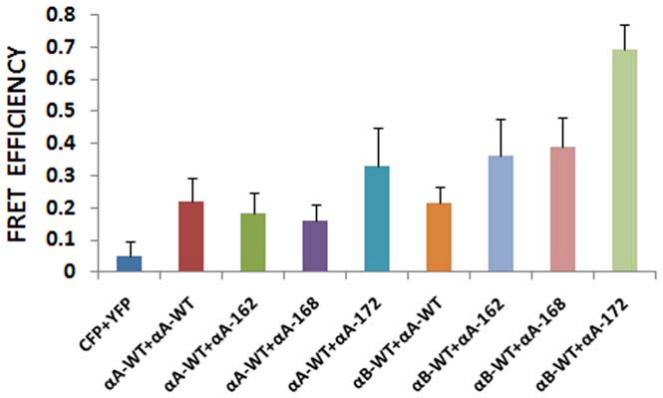
Bar diagram indicating the level of FRET efficiency. FRET efficiency demonstrates the interaction between the αA and αB subunits of α-crystallin. The interaction was strong between the wild types of αA and αB subunits. The interaction between the truncated constructs, αA162 and αA168 with αAwt and αBwt were lower compared to αA172 expression. The results are expressed as mean ± Standard Deviation (SD).

The acceptor photo-bleaching method was used to determine the intensities of interactions (FRET efficiency) of the C-terminal truncated αA-crystallins with αAwt and αBwt. It is expected that when the acceptor fluorescence is completely bleached the donor fluorescence intensity increases proportionately and this increase is considered a measure of the interaction between the two proteins. Co-expression of CFP and YFP vectors only followed by photo-bleaching of the acceptor YFP showed no increase in the donor CFP fluorescence intensity which is indicative of the lack of interactions between the vectors alone. Pre-bleach and post-bleach LSM images of CFPαAwt/YFPαA-truncated and CFPαBwt/YFPαA-truncated showed complete or nearly complete photo-bleaching; Fig. 7 illustrates, as an example, photo-bleaching of YFPαA172 (acceptor) and increase in fluorescence intensity of CFPαBwt (donor). FRET efficiency values were generated from LSM images, calculated as discussed in ‘Methods’. These values were generated for homologous interactions where αAwt interacts with C-terminal truncated αA-crystallins and also for heterologous interactions where αBwt interacts with C-terminal truncated αA-crystallins (Fig. 8) As expected, negative control (vectors alone) showed very little interaction while positive controls (αAwt/αAwt and αBwt/αAwt) showed significant interaction. However, in αAwt/αA162 and αAwt/αA168 FRET efficiencies were nearly 30% lower than that of αAwt/αAwt whereas in αAwt/αA172 FRET efficiency was 50% higher. CFPαBwt/YFPαAwt and CFPαBwt/YFP-truncated αA-crystallins also showed complete photo-bleaching. FRET efficiency in αBwt/αA168 was slightly higher and in αBwt/αA172 two-fold higher than in αBwt/αAwt. Moreover, the overall interaction of the C-terminal truncated αA-crystallins with αBwt was two-fold higher than with αAwt.

### Detection of Aggresomes in truncated αA-crystallin expressed cells

**Figure 9:**
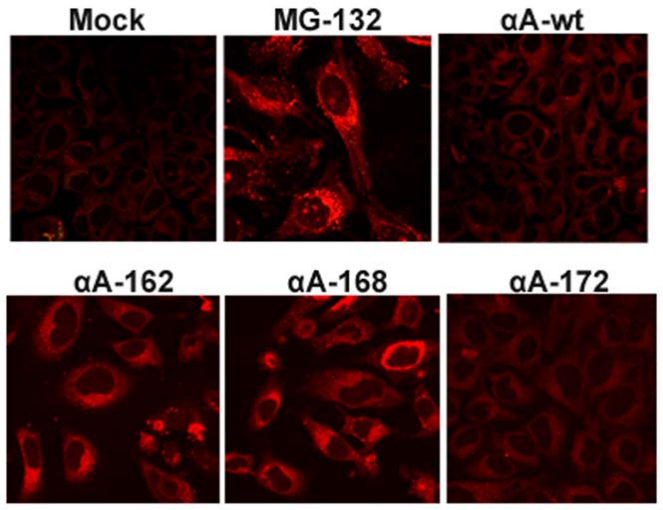
Detection of Aggresomes in individual expression of truncated αA in HeLa cells. Bright punctate staining in the perinuclear region for aggresomes was evident in αA162 and αA168 individually expressed cells. The proteasomal inhibitor, MG132 was used as positive control which exhibited a dramatic increase in punctate fluorescent staining in the perinuclear or juxta-nuclear region of the microtubule organizing center (MTOC) of the cells, and there was no aggresomes in negative control cells treated with vehicle, DMSO. Aggresomal foci were not evident in either αAwt or αA172 expressed cells.

**Figure 10:**
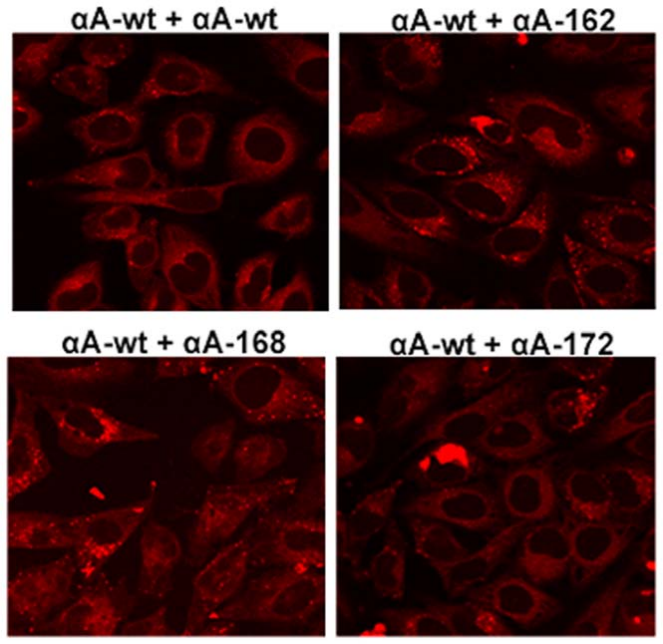
Aggresomes detected in YFP-tagged truncated constructs co-expressed with CFPαAwt. Aggresome foci were still visible in αAwt co-expressed only with αA162 and αA168 constructs but not in co-expression with αAwt and αA172 constructs.

**Figure 11:**
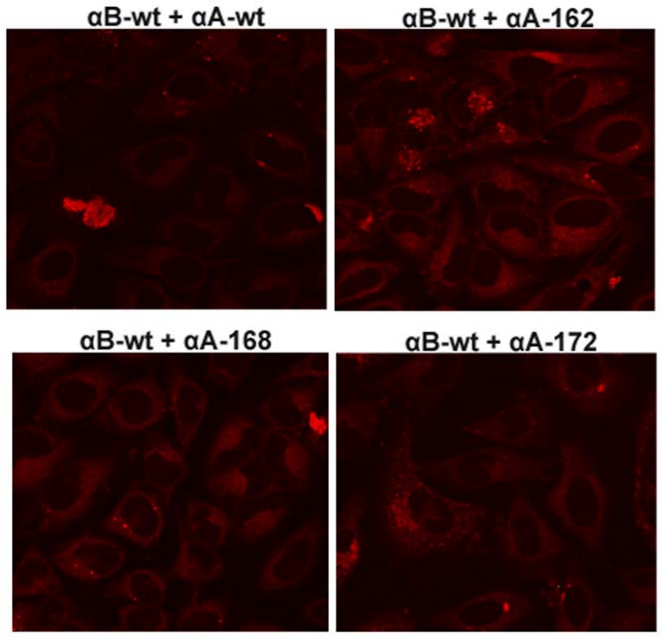
Aggresomes were not detected in CFPαB-wt co-expressing cells. There were no aggresome foci in αBwt co-expressed cells with wild-type and the truncated constructs. The images are representative of four such images obtained in three independent experiments.

Aggresomes are known to serve as storage bins of misfolded or aggregated proteins. Since the truncated αA-crystallin forms intracellular aggregate, we sought to investigate whether truncated αA-crystallin expression forms aggresomes, we stained the cells with Proteostat Aggresome dye after 48 hour transfection. This dye has been used to detect misfolded and aggregated proteins within aggresomes and inclusion bodies in cells [23]. Interestingly, bright punctate staining for aggresomes localized specifically in the perinuclear or juxta-nuclear sites of the cells individually expressing αA162 and αA168 but not in αAwt and αA172 and these results is very similar with the positive control where cells treated with a potent cell-permeable proteasome inhibitor, MG-132 (5 µM) for 15 hours. (Fig. 9). There was no decrease in the number of cells containing aggresomes in co-expression of αA-wt with αA162 and αA168 constructs (Fig. 10). In contrast, co-expression of αB-wt significantly diminished the aggresome formation in cells expressing with αA162 and αA168 constructs (Fig. 11).

**Figure 12:**
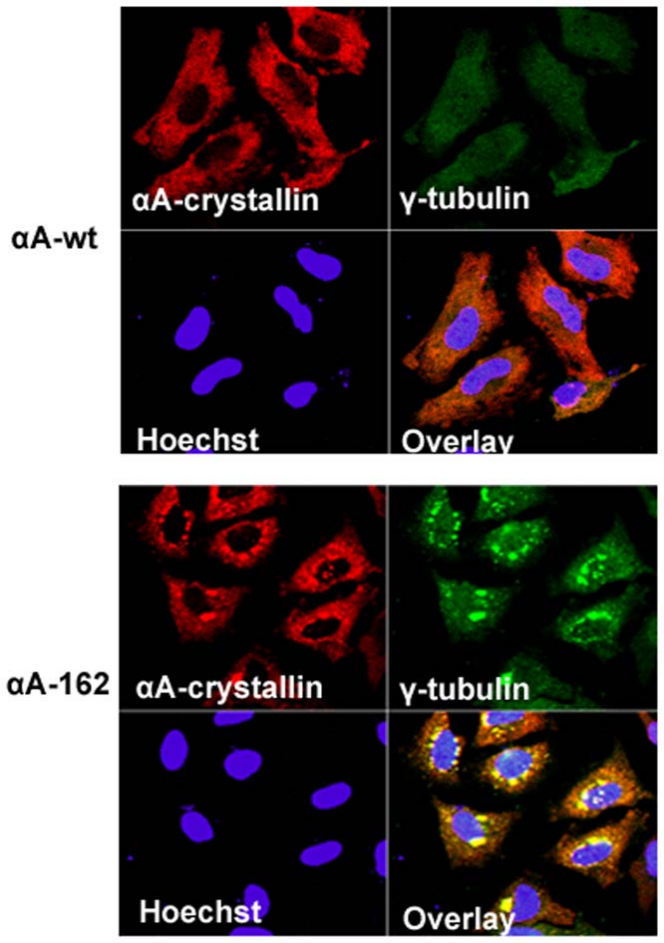
Inclusion bodies containing truncated αA-crystallin are aggresomes. After 48 hours transfection, cells were double immunostained with αA-crystallin (Red) and γ-tubulin (green). The co-localized (yellow) perinuclear signals characterize positive for aggresomes only in the truncated αA162 expressing cells. γ-tubulin did not co-localize in cells expressed with αAWT. Cells were counterstained with Hoechst 33342, allowing the detection of nuclei (blue).

**Figure 13:**
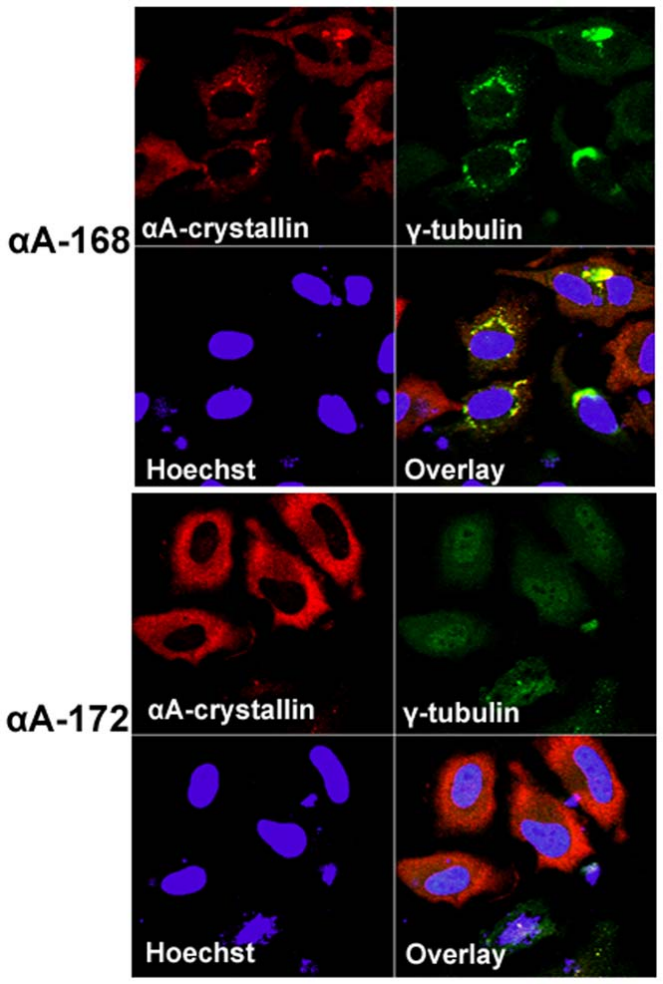
Validation for aggresomes in HeLa cells transfected with YFP-αA168 and YFP-αA172 constructs. 48 hours post-transfection cells were double immunostained with αA-crystallin (Red) and γ-tubulin (Green) antibodies. The γ-tubulin did not co-localize in YFPαA-172 expressing cells, but co-localization signal was evident in YFP-αA168 expressing cells. Nuclei were stained with Hoechst 33342 (blue).

To further validate the perinuclear inclusions as αA-crystallin-positive aggresomes as judged by staining with ProteoStat Aggresome dye, transfected cells were subjected to double immunostaining with αA-crystallin and γ-tubulin antibodies. The γ-tubulin has been previously shown to co-localize with aggresomes in the microtubule organizing center (MTOC) [24]–[27]. We compared the αA-crystallin and γ-tubulin staining in the transfected cells and our results suggest a strong degree of overlap in staining the perinuclear region. The data is consistent with the interpretation of only the two truncated versions of αA-crystallins, αA162 and αA168 form aggresomes but not in cells expressing with αAwt and αA172 (Fig. 12 and Fig. 13).

## Discussion

In the present study, we have studied various CFP and YFP tagged α-crystallins expression in HeLa cells. Human lens epithelial cells may have been a preferable choice, however, the level of expression of αA- crystallin and αB-crystallin is expected to be too low in the available human epithelial cell lines to obtain significant LSM signal. Moreover, the epithelial cells are known to have endogenous αB-crystallin and it would have complicated the study. All the three truncated αA-crystallins investigated in this study showed various extent of protein aggregation when each construct was transfected in HeLa cells, either individually or with αAwt or αBwt, for 48 hours (Fig. 1, 3, & 5). It was not possible to ascertain the actual level of aggregated protein in individual cells, instead, depended solely on visual assessment. There is no accurate method is available to quantify the intracellular protein aggregations in the cells. We used Dynamic Light Scattering (DLS) assay to measure the aggregations from the lysed samples, but we do not see any light scattering in these samples. The reason is DLS technology will not able to track the aggregation of protein of interest when more than one protein present in the lysed sample. This makes sense, because the aggregates form a high molecular weight complex and also the size of the particles in the complex overlaps with the other particles present in the sample. DLS technique is often applied to aggregation studies on purified proteins which is 95% purity or higher. In fact, the LSM images provided in figures 1A, 3A, and 5A give the true appearance of the living HeLa cells with different levels of aggregates.

The question arises as to what makes the truncated αA-crystallins to aggregate in mammalian cells. Lack of protein stability and conformational changes could be two major factors that could influence aggregation. In an earlier study, we have tested the stability of the truncated αA-crystallins at 25 and 37°C by measuring light scattering for 30 minutes [17]. All the three truncated αA-crystallins were stable at 25°C and only αA162 was slightly unstable at 37°C. However, all the three truncated αA-crystallins, were under different degree of unfolding stress when they were expressed individually for 48 h in HeLa cells. As homoaggregates, while they were not associated with αAwt or αBwt, they exhibited conformational changes, αA162 showing the largest change [17]. Moreover, αA162 showed three-fold increase in the α-helical content accompanied by a loss in β-sheet conformation and an increase in random coil conformation [17]. Thus, it appears that the conformational changes make these truncated αA-crystallins aggregation prone. Association with αAwt did not prevent their susceptibility to aggregation (Fig. 3). However, interaction with αBwt significantly diminished the aggregation of each of the truncated αA-crystallin, αA168 and αA172 showing the most effect and αA162 showing the least effect (Fig. 5). αB-crystallin is known to be a better molecular chaperone than αA-crystallin and it readily recognizes partially unfolded structures and prevent them from aggregation. Structural studies suggest that αA172 and αA168 are partially unfolded [17] and, so, αB-crystallin binds effectively to these polypeptides and the aggregation process is nearly completely prevented in αA172 and significantly decreased in αA168. In the case of αA162, there is strong evidence for the presence of fully unfolded structural entities [17] which explains why αB-crystallin failed to recognize them and, thus, unable to completely prevent protein aggregation.

Earlier *in vitro* FRET studies performed in our laboratory have confirmed C-terminal truncated αA-crystallins having weak interactions with both αAwt and αBwt [18]. This was exceptionally true for αA162 interacting with αBwt because the subunit exchange rate (k) was 0.65×10^−4^ S^−1^ as compared to 4.1×10^−4^ S^−1^ for αAwt interacting with αBwt [18]. The decreased interaction of the truncated αA-crystallins with αAwt or αBwt is expected to increase the probability of the truncated αA-crystallins existing as homoaggregates rather than heteroaggregates. This may put these truncated αA-crystallins in aggregation mode in the cell. Small heat-shock proteins (sHsps) like αA- and αB-crystallins may also operate by a different mechanism by which preformed protein aggregates are dissociated first by sHsps for subsequent re-folding by the Hsp70 chaperone machine. However, it is uncertain whether such a pathway exists in these cells.

Dysregulation of the degradation of misfolded and aggregated proteins or the protein quality control pathway has been implicated in Cystic Fibrosis, many neurodegenerative diseases and cancer [28]–[31]. In the present study, we have used a dye, ProteoStat Aggresome dye for the detection of aggresomes. This dye has been used to detect the aggregated proteins and peptides within aggresomes and related inclusion bodies in cells and tissues [23]. Furthermore, this dye is non-fluorescent in solution but becomes brightly fluorescent upon binding to the tertiary structure of aggregated proteins [23]. Formation of aggresomes in the truncated constructs, αA162 and αA168, suggests that it may be a cellular response and the inhibition of aggresomes in C-terminal truncated constructs co-expressed with αBwt strongly suggests that chaperones are able to refold the aggregated proteins. Moreover, there was no sequestration of aggresomes in αA-wt co-expression with these truncated constructs suggest that it co-aggregates with the unfolded protein products of the αA162 and αA168 and localized into the perinuclear sites of the cells. Our results on formation of aggresomes were similar with another report on a myopathy-causing αB-crystallin mutant, R120G forms aggresomes in cell culture models [32].

It has been reported that multiple aggregates or pre-aggresome particles may be an intermediate step in aggresome formation which can proceed further upon inhibition of proteasome [33]. The present study documented both multiple aggregates and typical perinuclear localized aggresomes in HeLa cells over-expressing the C-terminal truncated αA-crystallin genes. Aggresomes are special protective structures that fundamentally differ from other multiple aggregates, that some of which cause cellular toxicity. They are formed around centrosome/microtubule organizing center (MTOC), a sub-cellular region is robustly enriched with chaperones and components of UPS. [19]. Indeed it has been reported that there is a close correlation between aggresome formation and cell survival. [34]. More studies are needed in this direction to elucidate the role of aggresomes and the signaling pathway in diseases associated with αA-crystallin mutants.
